# Macaque Homologs of EBV and KSHV Show Uniquely Different Associations with Simian AIDS-related Lymphomas

**DOI:** 10.1371/journal.ppat.1002962

**Published:** 2012-10-04

**Authors:** A. Gregory Bruce, Helle Bielefeldt-Ohmann, Serge Barcy, Angela M. Bakke, Patrick Lewis, Che-Chung Tsai, Robert D. Murnane, Timothy M. Rose

**Affiliations:** 1 Seattle Children's Research Institute, Seattle, Washington, United States of America; 2 University of Queensland, Gatton, Queensland, Australia; 3 University of Washington, Seattle, Washington, United States of America; 4 Northwestern University, Evanston, Illinois, United States of America; 5 Washington National Primate Research Center, Seattle, Washington, United States of America; University of Southern California Keck School of Medicine, United States of America

## Abstract

Two gammaherpesviruses, Epstein-Barr virus (EBV) (*Lymphocryptovirus* genus) and Kaposi's sarcoma-associated herpesvirus (KSHV) (*Rhadinovirus* genus) have been implicated in the etiology of AIDS-associated lymphomas. Homologs of these viruses have been identified in macaques and other non-human primates. In order to assess the association of these viruses with non-human primate disease, archived lymphoma samples were screened for the presence of macaque lymphocryptovirus (LCV) homologs of EBV, and macaque rhadinoviruses belonging to the RV1 lineage of KSHV homologs or the more distant RV2 lineage of Old World primate rhadinoviruses. Viral loads were determined by QPCR and infected cells were identified by immunolabeling for different viral proteins. The lymphomas segregated into three groups. The first group (n = 6) was associated with SIV/SHIV infections, contained high levels of LCV (1–25 genomes/cell) and expressed the B-cell antigens CD20 or BLA.36. A strong EBNA-2 signal was detected in the nuclei of the neoplastic cells in one of the LCV-high lymphomas, indicative of a type III latency stage. None of the lymphomas in this group stained for the LCV viral capsid antigen (VCA) lytic marker. The second group (n = 5) was associated with D-type simian retrovirus-2 (SRV-2) infections, contained high levels of RV2 rhadinovirus (9–790 genomes/cell) and expressed the CD3 T-cell marker. The third group (n = 3) was associated with SIV/SHIV infections, contained high levels of RV2 rhadinovirus (2–260 genomes/cell) and was negative for both CD20 and CD3. In both the CD3-positive and CD3/CD20-negative lymphomas, the neoplastic cells stained strongly for markers of RV2 lytic replication. None of the lymphomas had detectable levels of retroperitoneal fibromatosis herpesvirus (RFHV), the macaque RV1 homolog of KSHV. Our data suggest etiological roles for both lymphocryptoviruses and RV2 rhadinoviruses in the development of simian AIDS-associated lymphomas and indicate that the virus-infected neoplastic lymphoid cells are derived from different lymphocyte lineages and differentiation stages.

## Author Summary

The incidence of Kaposi's sarcoma (KS) and non-Hodgkin's lymphoma increased in conjunction with the epidemic of HIV disease and AIDS. These malignancies are now known to be associated with secondary infections with a gammaherpesvirus; KS, with the Kaposi's sarcoma-associated herpesvirus (KSHV) and lymphoma, with both KSHV and Epstein-Barr virus (EBV). Similar AIDS-related malignancies have been observed in monkeys with simian AIDS and monkey gammaherpesviruses related to KSHV and EBV have been implicated in the development of disease. The study of monkey models of AIDS-related malignancies provides important approaches for understanding the role of gammaherpesviruses in AIDS-related tumorigenesis. Here we have used a combined molecular and immunological approach to identify, quantitate and localize infections of gammaherpesviruses in AIDS-associated lymphomas in macaques. We found high levels of macaque viruses related to EBV and KSHV in the tumor cells of distinct types of macaque lymphomas, suggesting that the virus-infected tumor cells belong to different lymphocyte lineages and differentiation stages.

## Introduction

Members of the gammaherpesvirus subfamily have been implicated in the etiology of a variety of malignancies [1]. Epstein-Barr virus/human herpesvirus 4 (EBV), genus *Lymphocryptovirus* (LCV), has long been associated with the development of B-cell lymphoproliferative disorders, including Burkitt's lymphoma, Hodgkin's lymphoma, post-transplant and HIV-associated lymphoproliferative disorders, and is also associated with epithelial-derived tumors, including nasopharyngeal and gastric carcinomas [2]. The related gammaherpesvirus, Kaposi's sarcoma-associated herpesvirus virus/human herpesvirus 8 (KSHV), genus *Rhadinovirus* (RV), is the etiological agent of Kaposi's sarcoma (KS), an endothelial cell derived malignancy [3]. In addition, KSHV plays a role in the pathogenesis of two rare B-cell lymphoproliferative disorders, primary effusion lymphoma (PEL) and multicentric Castleman's disease (MCD/MCD-associated plasmablastic lymphoma), and is associated with HIV-related solid immunoblastic/plasmablastic diffuse large B-cell lymphoma [4]. In some cases, including HIV-associated PEL, the B-cell tumors can be co-infected with both EBV and KSHV [5]. In rare cases, KSHV and EBV have been detected in T-cell lymphoproliferative disorders [6], although an etiologic role has not been established.

In malignancies associated with either KSHV or EBV infection, the vast majorities of tumor cells are latently infected and contain only a restricted number of viral episomes. The spindeloid tumor cells in KS lesions contain 1–2 KSHV genomes per cell [7] and express the latency-associated nuclear antigen (LANA), indicative of a latent phenotype [8], [9]. Only a small number of tumor cells are reactive with antibodies to the KSHV DNA polymerase processivity factor, ORF59, a marker of virus replication [10]. In nasopharyngeal carcinoma, diffuse large cell lymphoma and AIDS-associated lymphoma, the EBV load ranges from 1–14 EBV genomes/cell [11]. Similarly, EBV-positive tumors show various latency programs of infection defined by the differential expression of the EBV nuclear antigens (EBNAs 1,2,3A, 3B, 3C and LP), the small non-coding RNAS, EBER1 and EBER2, and the latent membrane proteins (LMPs 1, 2A and 2B) [2]. The detection of high levels of viral genomes by qPCR and concomitant expression of virus-specific proteins in the neoplastic cells provides strong evidence for an etiologic role of KSHV and EBV in tumorigenesis.

**Figure 1:**
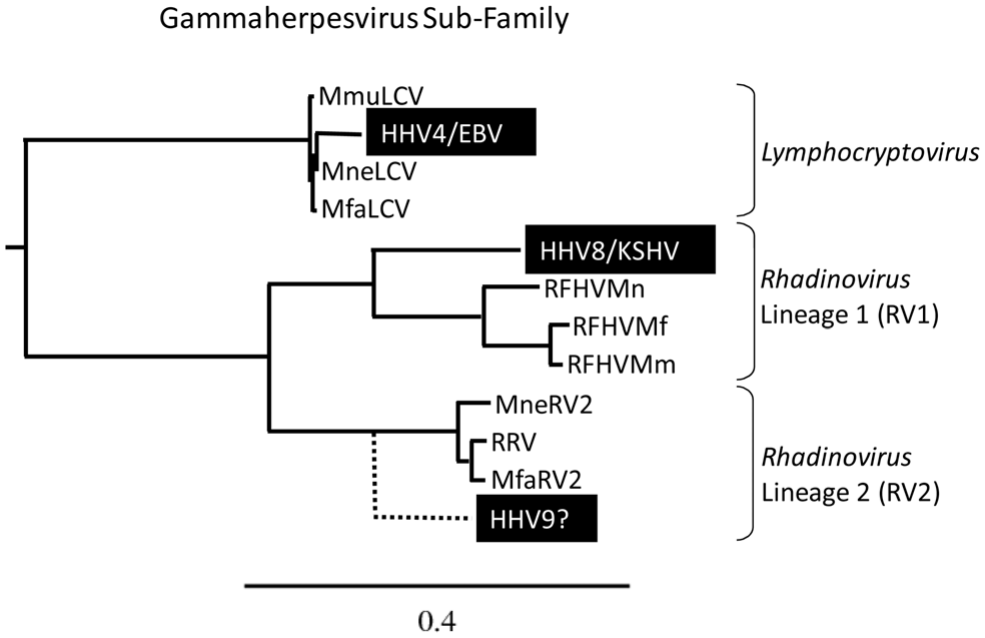
Phylogenetic relationship of the gammaherpesvirus subfamily. The DNA polymerase sequences of known human (black highlight) and macaque gammaherpesviruses were analyzed by maximum likelihood. *Lymphocryptovirus* genus: Human herpesvirus 4/Epstein-Barr virus (HHV4/EBV; YP_401712) and macaque lymphocryptovirus of *M. mulatta* (MmuLCV; AAK95475), *M. nemestrina* (MneLCV; unpublished data) and *M. fascicularis* (MfaLCV; AF534221); *Rhadinovirus* genus, RV1 lineage of Old World primate rhadinoviruses: human herpesvirus 8/Kaposi's sarcoma-associated herpesvirus (HHV8/KSHV; AAC57086) and macaque retroperitoneal fibromatosis herpesviruses of *M. mulatta* (RFHVMm; AAC57976), *M. nemestrina* (RFHVMn; AAF81662), and *M. fascicularis* (RFHVMf; AAN35122); and the RV2 lineage of Old World primate rhadinoviruses: macaque RV2 rhadinoviruses of *M. mulatta* (RRV; NP_570750), *M. nemestrina* (MneRV2; unpublished data), and *M. fascicularis* (MfaRV2; ABU52895). LCV, RV1 and RV2 gammaherpesviruses have been identified in other New and Old World non-human primates, including chimpanzee and gorilla (not shown). Although the existence of a novel RV2 rhadinovirus in humans (HHV9?) is predicted from an evolutionary perspective [15], [16], the virus has not yet been identified. The phylogenetic clustering of the rhesus and cynomolgus macaque rhadinoviruses reflects the close evolutionary relationship of their primate hosts [51]. The substitutions per site are indicated.

Close phylogenetic relationships have been identified between human and non-human Old World primate gammaherpesviruses (see Figure 1). Lymphocryptoviruses closely related to EBV have been identified in rhesus (RhLCV/MmuLCV) [12], pig-tailed (HV(mne)/MneLCV) [13] and cynomolgus (HVMF-1/MfaLCV) [14] macaques, and other primate species. Two distinct lineages of KSHV-related rhadinoviruses have been identified in macaques and other non-human Old World primates [15], [16]. The RV1 rhadinovirus lineage consists of KSHV and closely related homologs in macaques, gorillas, chimpanzees and other Old World primates. Retroperitoneal fibromatosis-associated herpesvirus (RFHV), the RV1 rhadinovirus of macaques, was identified in AIDS-related KS-like tumors in rhesus, pig-tailed and cynomolgus macaques [17]. The RV2 rhadinovirus lineage consists of more distantly related rhadinoviruses identified in macaques and other Old-World primates. While rhesus macaque rhadinovirus (RRV) is the prototype RV2 rhadinovirus [18], RV2 rhadinoviruses have been characterized in other macaque species, including pig-tailed (MneRV2) [15] and cynomolgus (MfaRV2) macaques [19] (Figure 1). The existence of a human RV2 rhadinovirus is predicted from an evolutionary perspective, but has not yet been confirmed [15], [20].

The macaque LCV and RV1 and RV2 rhadinoviruses are highly prevalent and persistent in adult animals with infection rates reaching 90% [18], [21], [22]. Like their human counterparts, the macaque gammaherpesviruses have been implicated in the etiology of a number of AIDS-associated malignancies [23]. However, the near ubiquity of these viruses in macaque populations has complicated studies on their role in tumor development. As a large percentage of animals show evidence of co-infection of LCV and the RV1 and RV2 rhadinoviruses, quantitative PCR (QPCR) techniques are required to distinguish tumor-associated virus from the levels of incidental virus in non-tumor cells. This analysis is complicated by the overall increase of incidental virus levels in non-tumor tissue following the course of immunosuppression during SIV or other immunodeficiency virus infections [24]. In these cases, immunosuppression not only leads to tumor induction and proliferation of virus-infected tumor cells, but also to proliferation and dispersal of virus in non-transformed cells.

Lymphomas are relatively rare in macaques, although an increased incidence has been noted in animals experimentally infected with simian immundeficiency virus (SIV) [25]. Previous studies have examined the association of macaque LCV with lymphoma in SIV-infected animals. In most studies, LCV was detected in nearly all the lymphomas, suggesting an etiological association [24], [26]–[32]. However, in nearly half of the cases analyzed, only low levels of LCV DNA were detected and few tumor cells stained positive for LCV antigens. This raises the question of whether the low virus load was due to incidental LCV infection of non-tumor cells present in the tumor lesion.

In contrast to the numerous studies on the prevalence of macaque LCV in SIV-associated lymphomas, only a few studies have examined the prevalence of macaque rhadinoviruses in lymphomas. QPCR was used to quantitate the viral load of the RV2 rhadinovirus prototype RRV in 19 lymphomas from SIV-infected rhesus macaques [33]. RRV DNA was detected in only one-half of the lymphoma samples with a median viral load of one virus per 2,600 cells. This study concluded that while prevalent in the macaques with lymphomas, RRV was rare within the lymphoma mass, most likely present incidentally in infiltrating non-tumor cells. Two of the animals showed lymphoid hyperplasia that is frequently detected in SIV-infected macaques. RRV DNA was only detected in one of these lesions, at a viral load of one virus per 540 cells, indicating that hyperplasia was not due to accumulation of RRV-infected cells. Another group reported that lymphomas were detected after experimental RRV infection in 1 of 15 SIV-infected rhesus macaques and 1 of 2 macaques without SIV [34]. By PCR analysis, the lymphomas were negative for LCV and RFHV, but positive for RRV, however the viral load was not quantitated.

In the present study, we examined archived lymphomas from the Washington National Primate Center (WaNPRC) for the presence of macaque gammaherpesviruses using QPCR assays to quantitate viral loads of LCV and RV1 and RV2 rhadinoviruses in tissue from different macaque species. We also used immunohistochemical approaches with antibodies to various viral proteins to determine whether the tumor cells were infected and whether the virus was in a latent or replicative stage. Our data supports an etiological role for both macaque lymphocryptovirus and RV2-lineage rhadinoviruses in SAIDS-associated lymphomas in multiple macaque species.

## Results

### Lymphoma cases

To study the role of gammaherpesviruses in the development of lymphomas in macaques, we examined archived lymphoma cases at the WaNPRC (summarized in Table 1). Five lymphoma cases occurred in the pig-tailed macaque breeding colony in the early 1980's during a colony-wide epidemic of the highly infectious D-type simian retrovirus-2 (SRV-2). In most cases, these animals were euthanized for clinical reasons other than the presence of a neoplasm, and lymphoma was diagnosed at necropsy. Subsequently, isolated lymphoma cases occurred in experimental study animals in AIDS-research protocols. These macaques had been infected with different strains of SIV or SIV/HIV hybrid viruses (SHIV). The majority of these animals were euthanized due to symptoms of simian AIDS with low CD4 count, and the lymphomas were diagnosed at necropsy. An additional lymphoma case was obtained from the Lovelace Respiratory Research Institute. This macaque (A07762) was euthanized during quarantine due to clinical disease and diagnosed with malignant lymphoma at necropsy. Finally, a DNA sample from an SIV-infected rhesus lymphoma case (L758) from the Tulane National Primate Research Center (TNPRC) was obtained. This lymphoma sample had previously been analyzed by QPCR for the presence of RRV [33].

**Table 1:** Summary of lymphoma cases.

Animal	Sp.^1^	Virus Status	Lymphoma Classification	Phenotype^2^				Viral genomes/million cells^3^		
				CD3	CD20	BLA36	IgM	RV1^4^	RV2^5^	LCV^6^
T76088	Mne	SRV2	Lymphoblastic	+	Neg^7^	Nd^8^	Neg	Neg	790,000,000	Neg
T76321	Mne	SRV2	Lymphoblastic	+	Neg	Nd	Nd	Neg	8,500,000	Neg
T80120	Mne	SRV2	Lymphoblastic	+	Neg	Nd	Nd	Neg	11,000,000	Neg
T81497	Mne	SRV2	Anaplastic L.C.^9^	+	Neg	Nd	Nd	Neg	28,000,000	Neg
T81228	Mne	SRV2	Anaplastic L.C.	+	Neg	Nd	Nd	Neg	130,000,000	Neg
02206	Mne	SHIV	Centroblastic	Neg	+	Nd	+	Neg	7,200^10^	56,000^10^
L758	Mmu	SIV	Immunoblastic	Nd	Nd	+	Nd	Neg	800	4,000,000
95017	Mfa	SIV	Not classified	Nd	Nd	Nd	Nd	Neg	700	25,000,000
A01110	Mmu	SIV	Immunoblastic	Neg	+	Nd	Neg	Neg	42,000	550,000
A08044	Mfa	SHIV	Not classified	Neg	+	+/−11	Neg	Neg	Neg	3,700,000
A07762	Mfa	Nd	Not classified	Nd	+/−	Nd	+	Neg	Neg	9,200,000
93034	Mfa	SHIV	Follicular L.C.	Neg	Neg	+	Neg	Neg	260,000,000	20,000
99091	Mfa	SIV	Lymphoblastic	Neg	Neg	Nd	+	Neg	110,000,000	5,300
A01112	Mmu	SIV	Immunoblastic	Neg	Neg	+	Neg	Neg	1,700,000	Neg

1 macaque species: Mne = *M. nemestrina*; Mmu = *M. mulatta*; Mfa = *M. fascicularis*;

2 see discussion in text;

3 Calculated by comparing Ct values obtained from viral QPCR assays and a QPCR assay targeting the single copy OSM gene (see Materials and Methods). OSM QPCR detects cellular DNA in all cells present in the tissue section, and does not necessarily reflect the number of tumor cells;

4 RV1 = Rhadinovirus 1 lineage in macaques: RFHVMn in *M. nemestrina*, RFHVMm in *M. mulatta*, RFHVMf in *M. fascicularis*;

5 RV2 = Rhadinovirus 2 lineage in macaques: MneRV2 in *M. nemestrina*, RRV in *M. mulatta*, MfaRV2 in *M. fascicularis*;

6 LCV = lymphocryptovirus in macaques: LCVMn in *M. nemestrina*, LCVMm in *M. mulatta*, LCVMf in *M. fascicularis*;

7 Neg = below the limit of detection;

8 Nd = Not determined;

9 L.C. = large cell;

10 majority of tumor sample necrotic;

11 +/− = heterogenous expression in the tumor.

Detailed pathology reports for the lymphoma cases are provided in supplemental material. The lymphomas were classified morphologically using the updated Kiel classification of non-Hodgkin's lymphomas [35]. Of the early SRV-2 associated lymphomas that occurred in pig-tailed macaques, two were classified as lymphoblastic (T76321, T80120), one as immunoblastic (T76088) and the remaining two as anaplastic large cell lymphomas with Reed-Sternberg-like cells (T81497, T81228) (Table 1). The lymphomas from pig-tailed, rhesus and cynomolgus macaques experimentally infected with immunodeficiency viruses were classified as lymphoblastic (99091), immunoblastic (A01110, A01112, and L758), centroblastic (02206), and follicular large cell (93034). The other lymphomas from macaques 92170, 95017, A08044 and A07762 were not classified.

### Viral load quantitation by QPCR

To determine viral loads in the lymphomas, DNA was extracted from paraffin-embedded formalin-fixed or frozen tissue and analyzed by QPCR for the macaque LCV homologs of EBV and the macaque RV1 and RV2 rhadinovirus homologs of KSHV. We have previously developed RV1 and RV2 QPCR assays that are highly efficient and specifically detect RV1 and RV2 rhadinoviruses in different macaque species [36], [37]. We have also developed a QPCR assay that is efficient with a high sensitivity and can detect LCV from different macaque species, including rhesus, pig-tailed and cynomolgus (see Materials and Methods). In parallel, a QPCR assay targeting the single copy cellular gene, oncostatin M (OSM) from humans and other Old World primates was used to quantitate cell number [36]. This allowed for the viral load per cell to be determined in parallel for each virus.

In all fourteen lymphoma samples, the levels of RV1 rhadinoviruses were below the limit of detection. In contrast, eight of the fourteen lymphoma samples had high levels of RV2 rhadinoviruses with viral loads ranging from 1.7×10^6^–7.9×10^8^ RV2 genomes per million cells, indicating a minimum of 2–800 viral copies/cell (Table 1). The other six lymphoma samples had high levels of LCV with viral loads ranging from 5.5×10^5^–2.5×10^7^ LCV genomes per million cells, indicating a minimum of 1–25 LCV copies/cell. In the eight lymphomas with high RV2 levels, the levels of LCV were either below the limit of detection (six cases) or 13,000–20,000 fold lower than the RV2 levels (two cases). In the six lymphomas with high LCV levels, the levels of RV2 were either below the limit of detection (two cases) or 10–35,000 fold lower than the LCV levels (four cases) (Table 1).

### Phenotyping of the lymphomas

#### CD3-positive T-cell lymphomas

**Figure 2:**
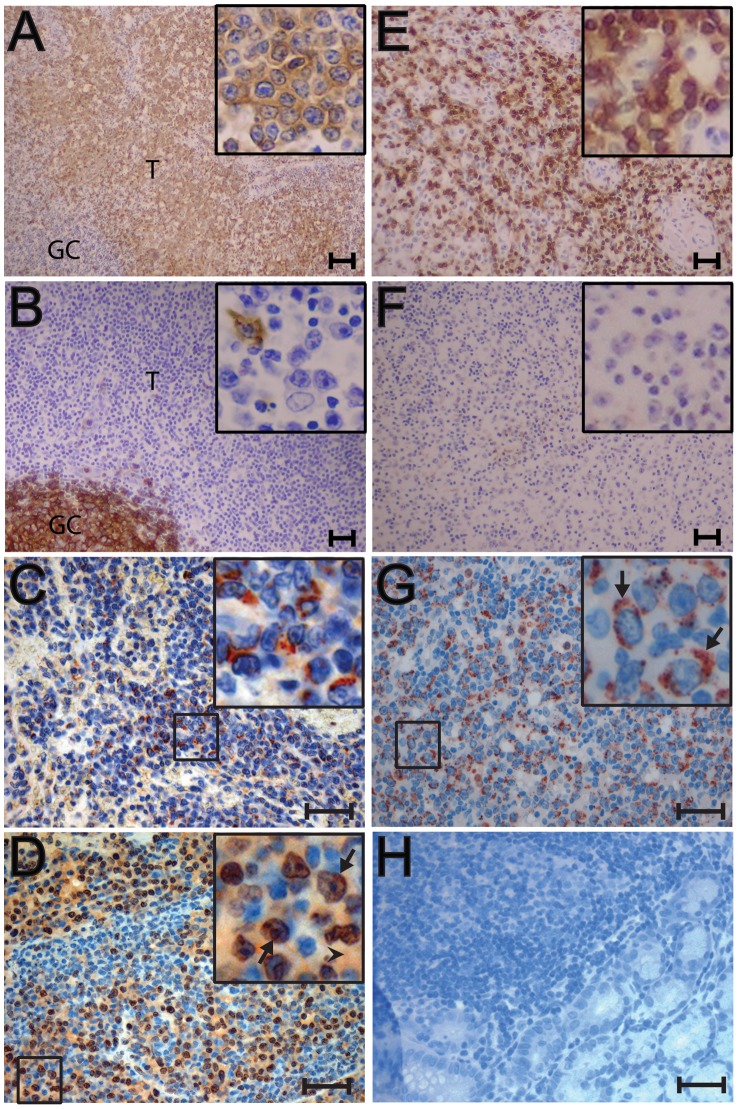
Immunohistochemical analysis of nodal and extranodal CD3-positive T-cell lymphomas of pig-tailed macaques. Metastatic lymphomas from an SRV2-positive/SIV-positive male pig-tailed macaque (T76321) (A–D) and an SRV2-positive female pig-tailed macaque (T76088) (E–G) were analyzed by immunostaining: A) anti-CD3 staining is evident in the lymphoblastic lymphoma (T) of T76321 surrounding a lymph node germinal center (GC). High magnification insert shows strong CD3 staining of large neoplastic cells with abundant cytoplasm, large nucleus and prominent nucleolus; B) anti-CD20 staining of T76321 tumor shown in (A). Strong CD20 staining of the germinal center (GC) cells is evident with a lack of staining in the surrounding tumor cells (T). High magnification insert shows negative staining for CD20 in the tumor cells with occasional CD20-positive cells; C) anti-gB (1508) staining of blastoid tumor cells within the lymph node of T76321. High magnification reveals strong cytoplasmic and paranuclear granular staining of blastoid cells with prominent nucleoli, possibly of the Golgi region; D) anti-ORF59 (425) staining of blastoid tumor cells invading the colon of T76321. The higher magnification reveals strong staining of the blastoid cell nuclei; arrows point to paranuclear and paranucleolar accumulation. Moderate staining of the blastoid cell cytoplasm is also detected (arrowhead). Staining is absent from the nuclei and cytoplasm of the smaller non-blastoid cells (blue nuclei); E) anti-CD3-staining of neoplastic cells within a visceral lymph node of T76088. High magnification insert shows strong CD3 staining of large neoplastic cells with abundant cytoplasm, a large nucleus with vesiculated chromatin and a prominent nucleolus; F) anti-CD20 staining of tumor shown in (E). Staining is absent in the tumor cells; G) anti-gB (1508) staining of tumor shown in (E). High magnification reveals strong cytoplasmic and paranuclear granular staining of blastoid cells with prominent nucleoli, possibly of the Golgi region; H) anti-gB (1508) staining of normal uninfected tissue. Non-specific staining is absent in all cell types. Scale bars = 75 µm (A, B, E, F); 37 µm (C, D, G, H). Panels C and D were autoleveled in Adobe Photoshop.

The lymphomas were phenotyped by immunohistochemical labeling for the CD20 B-cell marker and the CD3 T-cell marker. The five SRV-2 associated lymphomas from pig-tailed macaques phenotyped as CD3-positive T-cell lymphomas (Table 1). These macaques were diagnosed with multifocal lymphoma in multiple visceral organs. Two CD3-positive cases are shown in Figure 2. In T76321, lymphoblastic lymphomas were observed in spleen, liver, kidney, pancreas, lymph nodes, lung, adrenal gland, urinary bladder, salivary gland and bone marrow. Figure 2A shows a low magnification of CD3-positive lymphoma cells completely surrounding a CD3-negative lymph node germinal center. Higher magnification revealed the CD3-positive tumor cells to be large blastoid cells with one or two large nucleoli, dense dispersed chromatin and abundant cytoplasm (Figure 2A, inset). CD20 staining of the lymph node revealed strong staining of normal B-lymphocytes within the germinal center and only occasional CD20 staining of single cells in the peripheral area, which was filled with neoplastic CD3-positive lymphoid cells (Figure 2B). Similar strong CD3 staining was detected in the lymphoma cells invading the adrenal gland of this animal (data not shown). T76088 was diagnosed with multifocal lymphoma in lymph nodes and multiple visceral organs and tissues, including spleen, liver, kidney and lung. Figure 2E shows the CD3-positive neoplastic cells infiltrating a lymph node. The tumor cells were large with abundant cytoplasm and a large nucleus with prominent nucleoli (Figure 2E, inset). CD3-positive tumor cells infiltrated the interstitium of the kidneys and other organs (data not shown). CD20 staining of the tumor was negative (Figure 2F) with only occasional CD20-positive cells located amongst the sheets of neoplastic cells. The CD3 staining of T76321 and T76088 was representative of that observed in the other three SRV-2 associated lymphomas.

**Figure 3:**
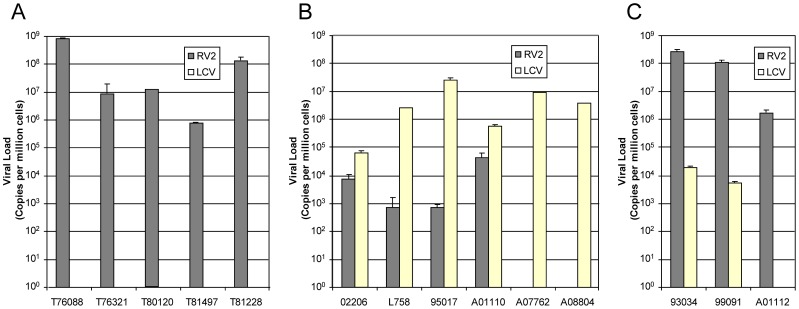
Viral loads of gammaherpesviruses in lymphomas from different macaque species. Viral loads were determined using QPCR assays that detect macaque lymphocryptoviruses (LCV) from rhesus (MmuLCV), pig-tailed (MneLCV) or cynomolgus (MfaLCV) macaques or macaque RV2 rhadinoviruses from the same macaque species (RRV, MneRV2 and MfaRV2, respectively) (see Table 1). The results were normalized to cell number in the tissue sample using a QPCR assay for oncostatin M, a single-copy cellular gene (see Materials and Methods). Viral load is expressed as genome equivalent copies per 10^6^ cells: A) CD3-positive T-cell lymphomas; B) CD20-positive B-cell lymphomas; C) CD3-negative/CD20-negative lymphomas.

All five of the SRV-2 associated T-cell lymphomas contained high levels of RV2 with a median level of 28 RV2 genomes/cell (range: 9–780) (Figure 3A). None of these lymphomas had detectable levels of LCV or RV1 rhadinovirus.

#### CD20 B-cell lymphomas

**Figure 4:**
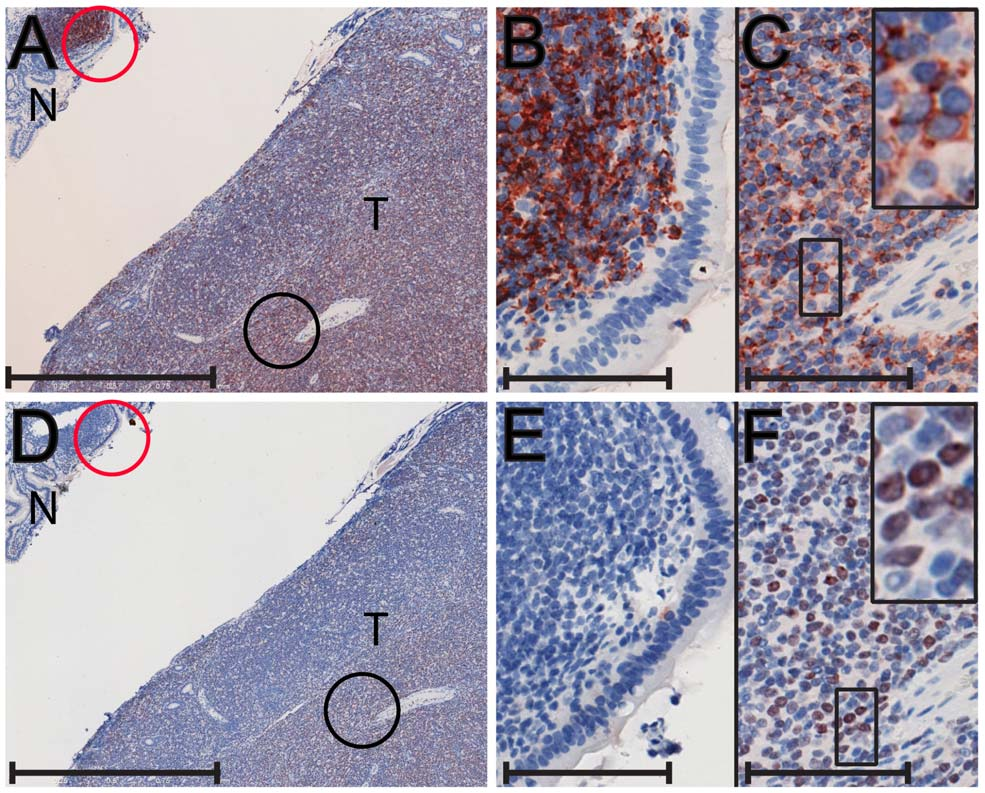
Immunohistochemical analysis of a CD20-positive B-cell lymphoma from the duodenum of a cynomolgus macaque. A metastatic lymphoma invading the duodenum of a SHIV-positive female cynomolgus macaque (A08044) was analyzed by immunostaining: A) anti-CD20 staining is evident in the lymphoma tumor (T) and in the normal (N) lymphoid follicle in the adjacent duodenum tissue; B) A higher magnification of the lymphoid follicle in (A), circled in red, revealed strong CD20 staining in the normal lymphoid cells; C) A higher magnification of the region in (A), circled in black, revealed strong CD20 staining in the large blastoid cells with prominent nucleoli in the tumor tissue; D) anti-EBNA-2 (LCV) staining is evident in the tumor region (T); E) A higher magnification of the normal follicle region in (D), circled in red, revealed an absence of EBNA-2 staining; F) A higher magnification of the tumor region in (D), circled in black, revealed strong EBNA-2 staining in the nuclei of most blastoid tumor cells. Scale bars = 1 mm (A,D); 100 µm (B,C,E,F).

Four of the CD3-negative lymphomas stained positive for the CD20 B-cell marker (Table 1). One CD20-positive case, A08044, is shown in Figure 4. A large abdominal mass was identified in this animal that adhered tightly to the cranial pole of the right kidney, the pancreas, the fundic and pyloric stomach, duodenum and proximal jejunum. Figure 4A shows a low magnification of the CD20-positive tumor (T) adjacent to CD20-negative epithelium of the duodenum (N) that surrounds a CD20-positive lymphoid follicle (red circle). Higher magnification revealed the lymphoid follicle to be composed of an aggregate of small dense CD20-positive lymphocytes that were adjacent to the elongated CD20-negative epithelial cells (Figure 4B). Additional CD20-positive lymphoid follicles were detected in the normal duodenum in this tissue sample (data not shown). Higher magnification revealed the tumor to be composed of large blastoid tumor cells with prominent nucleoli and abundant cytoplasm that were CD20-positive (Figure 4C). Similar CD20 staining was observed in the 02206 and A01110 lymphomas, while the A07762 lymphoma showed variable CD20 expression. Additional phenotyping was performed on selected lymphomas assaying for expression of the B-cell antigen BLA.36 and/or the early B-cell marker, IgM [38]. Two of the lymphomas stained positive for BLA.36 and two stained positive for IgM (Table 1).

In contrast to the CD3-positive T-cell lymphomas, which contained high levels of RV2 rhadinovirus and no detectable LCV, all four CD20-positive B-cell lymphomas contained high levels of LCV (Table 1 and Figure 3B). Two additional lymphomas from macaques 95017 and L758 also had high levels of LCV, but were not tested for CD20 reactivity due to lack of material to test. The median LCV load in all six of these lymphomas was nearly four LCV genomes/cell, while the RV2 level was at or below the level of detection in four of these lymphomas. The remaining two lymphomas had intermediate levels of RV2, ranging from 0.007–0.04 RV2 genomes/cell, which were approximately 10 fold lower than the levels of LCV.

#### CD3-negative/CD20-negative lymphomas

**Figure 5:**
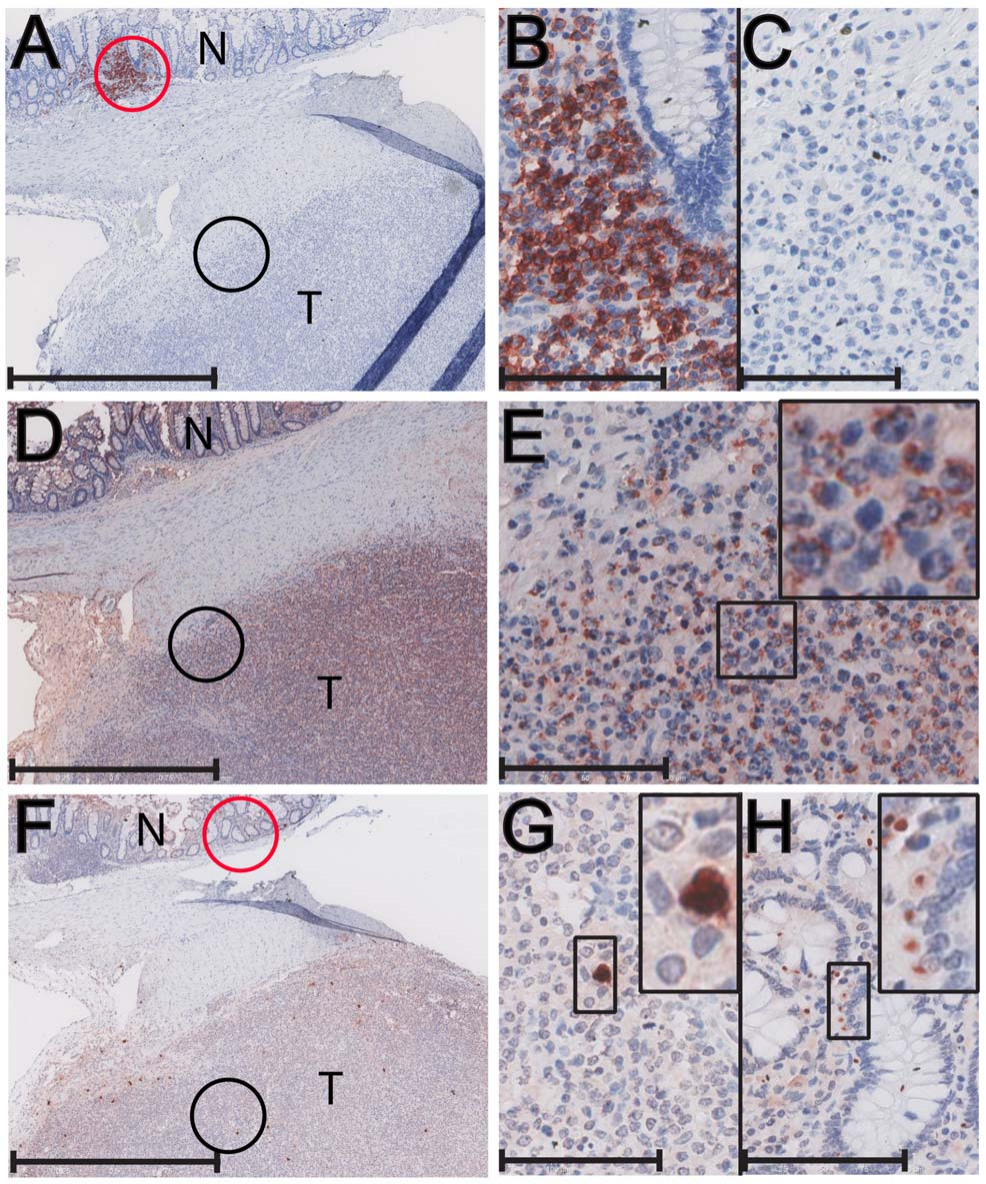
Immunohistochemical analysis of a CD20-negative/CD3-negative colonic lymphoma of a rhesus macaque. A metastatic lymphoma invading the colon of a SIV-positive male rhesus macaque (A01112) was analyzed by immunostaining: A) anti-CD20 staining is evident only in the normal lymphoid follicle (N); B) Higher magnification of the normal follicle in (A), circled in red, revealed strong CD20 staining in the normal lymphoid cells in the aggregate; C) Higher magnification of the tumor (T) region, circled in black, revealed a complete lack of CD-20 staining in the tumor cells; D) anti-gB (1508)(RV2) staining is evident in the tumor region (T); E) Higher magnification of the tumor region of (D), circled in black, revealed strong gB staining in essentially every blastoid tumor cell; F) anti-EBNA-2 (LCV) staining is evident in a few isolated cells in the tumor sample; G) Higher magnification of the tumor (T) region, circled in black, revealed strong nuclear and cytoplasmic EBNA-2 staining of only a few specific cells within the tumor; H) Higher magnification of the normal colonic epithelium in (F), circled in red, revealed discrete nuclear staining of specific cells flanking the epithelial cells in the mucosal crypts. Scale bars = 1 mm (A, D, F); 100 µm (B, C, E, G, H).

**Figure 6:**
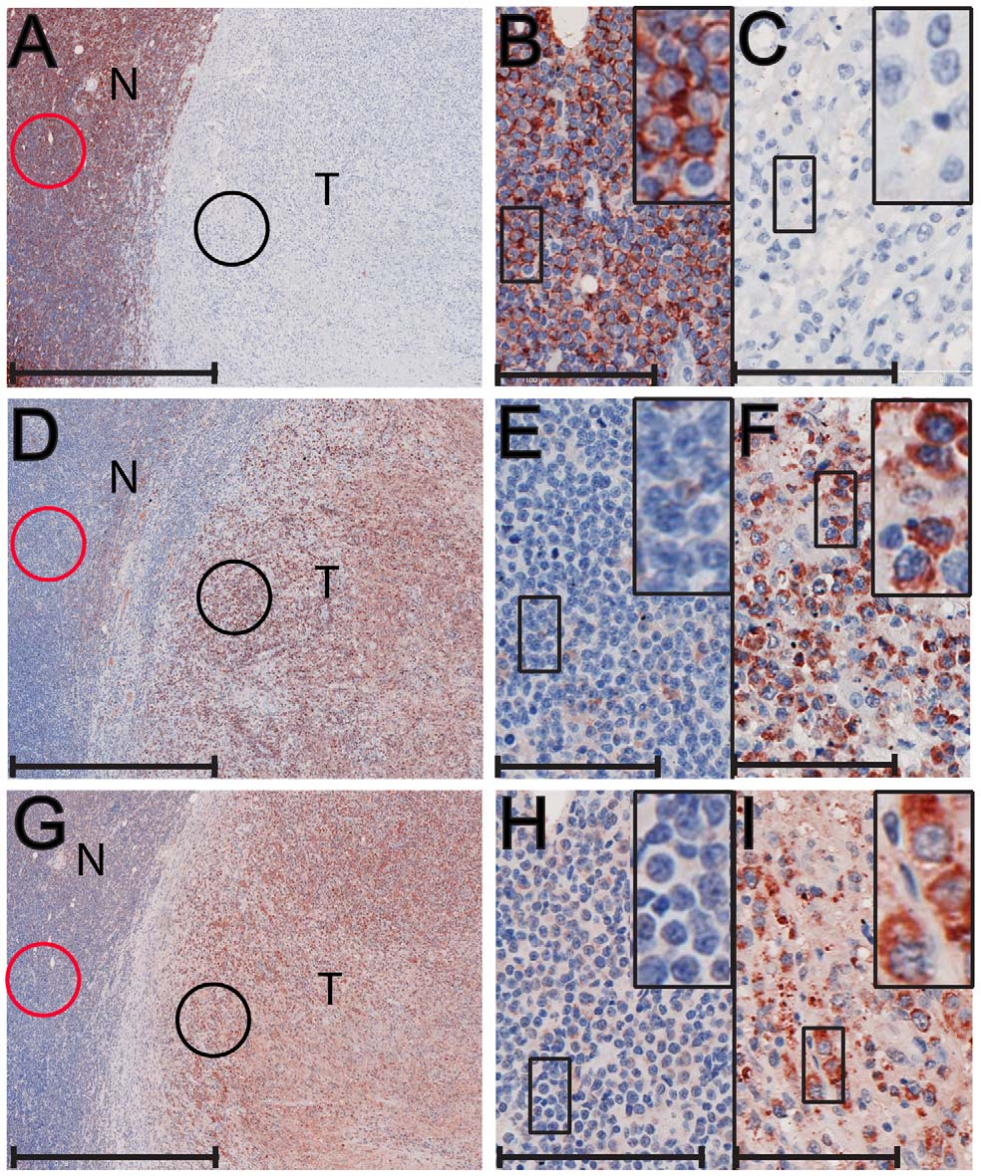
Immunohistochemical analysis of CD20-negative/CD3-negative adrenal lymphoma of a cynomolgus macaque. A metastatic lymphoma invading the adrenals of a SIV-positive male cynomolgus macaque (99091) was analyzed by immunostaining: A) anti-CD20 staining is evident only in the normal (N) lymphoid region surrounding the tumor (T); B) Higher magnification of the region in (A), circled in red, revealed strong CD20 staining in the normal lymphoid cells; C) Higher magnification of the tumor (T) region in (A), circled in black, revealed a complete lack of CD20 staining in the tumor cells; D) anti-IgM staining is evident in the tumor (T) region; E) Higher magnification of the CD20-positive lymphocyte region in (D), circled in red, revealed a lack of IgM staining in the normal lymphocytes; F) Higher magnification of the tumor region in (D), circled in black, revealed strong IgM staining of the tumor cells; G) anti-gB (1508)(RV2) staining is evident in the tumor region; H) Higher magnification of the CD20-positive lymphocyte region in (G), circled in red, revealed a lack of gB staining in the normal lymphocytes; I) Higher magnification of the tumor (T) region in (G), circled in black, revealed strong gB staining in essentially every blastoid tumor cell. Scale bars = 1 mm (A, D, G); 100 µm (B, C, E, F, H, I).

Three of the fourteen lymphomas (one rhesus and two cynomolgus macaques) did not stain specifically for either the CD20 B-cell marker or the CD3 T-cell marker. Two of these lymphomas are shown in Figures 5 and 6. Macaque A01112, which was infected with SIVsmE66, developed malignant lymphoma in multiple visceral organs including the lymph nodes, diaphragm, stomach and colon. Figure 5A shows a section of a tumor (T) from A01112 located adjacent to normal colonic mucosa (N) where small aggregates of normal small, dense B-lymphocytes, positive for CD20 were present (Figure 5A, red circle and Figure 5B). In contrast, neither the tumor mass nor the mucosal epithelium was positive for CD20 (Figure 5A). The tumor was composed of large blastoid cells with prominent nucleoli and abundant cytoplasm, which were negative for both CD20 and CD3 (Figure 5C, data not shown). Figure 6A shows a section of the lymphoma from 99091 presenting as a well-demarcated nodular growth that effaced the normal structure of an adrenal gland. Staining for CD20 was negative for cells within the tumor nodule (T) but positive for cells within the adjacent lymphoid tissue (N) (Figure 6A). Higher magnification showed that the tissue surrounding the tumor contained small densely-packed lymphocytes that stained strongly for CD20 (Figure 6B), while the tumor itself was composed of large blastoid cells with prominent nucleoli and abundant cytoplasm that were negative for both CD20 and CD3 (Figure 6C, data not shown). The other lymphoma (93034) showed a similar lack of reactivity for CD20 and CD3 (data not shown). The three lymphomas were also examined for IgM expression to determine whether they showed a pre-B cell phenotype. The 99091 lymphoma stained positive for the IgM pre-B cell marker. The CD20-positive normal lymphoid tissue surrounding the tumor mass of 99091 was negative for IgM expression (Figure 6D,E), whereas the tumor itself showed strong cytoplasmic expression of IgM (Figure 6D,F). The other two lymphomas from A01112 and 93034 were negative for IgM expression (Table 1).

All three of the CD3-negative/CD20-negative lymphomas contained high levels of RV2 rhadinovirus (Table 1 and Figure 3C), with a median viral load of 109 RV2 genomes/cell (range = 2–260). One of these lymphomas had no detectable LCV, while the other two showed evidence of a co-infection, with low LCV loads that ranged from 0.005–0.02 LCV genomes/cell (Table 1 and Figure 3C). The RV2 load in these lymphomas was 14,000–22,000 fold higher than the LCV viral load.

### Localization of viral antigens

The levels of RV2 viral DNA (up to ∼800 viral genomes/cell) detected in the CD3-positive and CD3-negative/CD20-negative lymphoma samples were significantly higher than the levels of RV1 viral DNA detected in previous studies of latent infections, both in KS tumors (∼2 KSHV genomes/cell) [7] and KS-like tumors in macaques (∼2 RFHV genomes/cell) [37], suggesting on-going RV2 replication in the lymphomas. In contrast, the load of LCV detected in the CD20-positive lymphoma samples (<25 LCV copies/cell) was similar to that detected in EBV latent infections in tumor tissue in vivo [11] and in infected tissue culture cells, such as Raji cells (unpublished observations). Therefore, to determine whether the viral load detected in the lymphoma samples reflected an actual infection of the malignant tumor cells, we performed immunohistochemical analysis using antisera that we have developed against the RV2 ORF59 DNA polymerase processivity factor [39] and the envelope glycoprotein B (gB) to detect markers of RV2 lytic replication, and a commercial monoclonal antibody to EBV EBNA-2, a marker of EBV latency. The RV2 ORF59 antiserum (425) reacts specifically with the ORF59 homologs of RRV, MneRV2 and MfaRV2, the RV2 lineage rhadinoviruses of rhesus, pig-tailed and cynomolgus macaques. This antiserum does not react with the RV1 ORF59 homologs of KSHV, RFHVMm, RFHVMn and RFHVMf or the ORF59 homolog of EBV [39]. The anti-gB serum (1508) is a pan RV1/RV2 antiserum that reacts with the virion envelope gB of both RV1 and RV2 rhadinoviruses (see Materials and Methods). The EBNA-2 monoclonal crossreacts with the LCV homologs of EBNA-2 and has been used previously to detect LCV infections in lymphomas in different macaque species [27], [28].

#### CD3-positive lymphomas

In the CD3-positive lymphoblastic lymphoma case (T76321), the 425 anti-RV2 ORF59 antiserum reacted with the nuclei of the blastoid tumor cells invading the colon (Figure 2D). The ORF59-positive nuclei were large with a characteristically prominent nucleolus (Figure 2D – insert, arrow). Specific paranuclear and paranucleolar ORF59 staining was evident. In many areas, obvious cytoplasmic staining was observed (Figure 2D – insert, arrowhead). The 1508 anti-gB antiserum also strongly reacted with the blastoid tumor cells within the lymph node of the same animal (Figure 2C). Higher magnification revealed cytoplasmic and paranuclear granular staining of gB with patchy accumulations (Figure 2C – insert). Figure 2G shows additional gB staining in blastoid tumor cells within a liver lymphoma of the CD3-positive immunoblastic lymphoma case (T76088). In these lymphoma samples, essentially every tumor cell showed evidence of gB expression. The strong staining of the tumor cells for both the nuclear ORF59 DNA polymerase processivity factor and the cytoplasmic/membrane-bound virion envelope gB coupled with the very high viral load (up to 800 viral genome copies per cell) strongly suggest an active replication of RV2 in these tumor cells. Similar expression of RV2 ORF59 and gB was observed in the other T-cell lymphomas confirming that the CD3-positive tumor cells were RV2-infected and were expressing both early and late markers of virus replication. Control experiments using tissue from an uninfected juvenile macaque that contained a variety of different cell types, including lymphoid and epithelial cells, revealed no non-specific binding of the 1508 anti-gB antisera (Figure 2H). Similar controls have shown a lack of non-specific binding of the 425 anti-RV2 ORF59 antisera [39], confirming the specificity of the observed antibody staining.

#### CD20-negative/CD3-negative lymphomas

**Figure 7:**
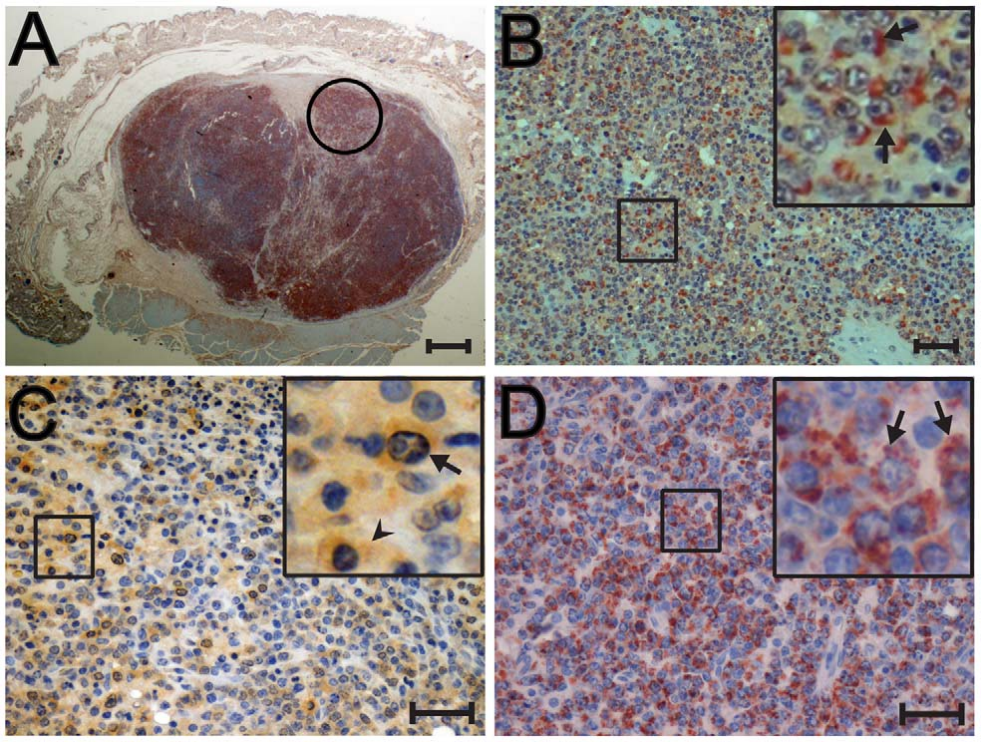
Immunohistochemical analysis of CD20-negative/CD3-negative cutaneous and muscle-associated lymphomas from rhesus and cynomolgus macaques. CD20-negative/CD3-negative lymphomas from a SHIV-positive female cynomolgus macaque (93034; cutaneous) (A,B) and a SIV-positive male rhesus macaque (A01112; abdominal muscle-associated lymphoma)(C,D) were analyzed by immunostaining: A) anti-gB (1508)(RV2) staining is evident in the entire cutaneous tumor of 93034 surrounded by displaced skin; B) High magnification of the region in A circled in black shows widespread expression of gB in the perinuclear area and cytoplasm; C) anti-ORF59 staining is evident in the majority of lymphoma cells in the A01112 lymphoma. High magnification of the boxed region shows strong nuclear, perinuclear and cytoplasmic ORF59 staining (insert); D) anti-gB (1508)(RV2) staining is evident in the majority of lymphoma cells in the A01112 lymphoma. High magnification of the boxed region shows strong staining in the cytoplasm and perinuclear regions, with an aggregate morphology. Figures 7A and C were autoleveled using Adobe Photoshop. Scale bars = 1 mm (A); 75 µm (B); 37 µm (C, D).

We next examined the CD20-negative/CD3-negative lymphomas that also showed high RV2 viral loads. Figure 7A shows the entire paraffin section from the 93034 cutaneous follicular large cell lymphoma stained with the 1508 anti-gB antiserum. The tumor mass constitutes the majority of the tissue sample and is surrounded by displaced skin. Essentially the whole tumor mass stained positive for gB. Figure 7B shows a magnification of the region circled in Figure 7A. As seen in this micrograph as well as in the magnified insert, strong gB staining was associated with essentially every blastoid tumor cell, showing the same patchy accumulations observed in the T-cell lymphomas. The 93034 lymphoma had the second highest RV2 viral load of nearly 300 viral genome copies per cell, showing a strong correlation between expression of the gB late lytic marker and high viral load. Figure 7C shows the RV2 ORF59 staining of blastoid tumor cells invading the lymph node in the CD20-negative/CD3-negative immunoblastic lymphoma case (A01112). As seen with the T-cell lymphomas, the neoplastic cells stained strongly for nuclear ORF59 with obvious paranuclear and paranucleolar staining (Figure 7C, insert, arrow). ORF59 cytoplasmic staining was also observed (Figure 7C – insert, arrowhead). Very strong anti-gB staining was observed in the A01112 lymph node lymphoma with staining associated with essentially every blastoid tumor cell (Figure 7D, insert). Figure 5D shows a section of colonic tumor from the same animal stained with the 1508 anti-gB antibody. Strong staining was detected in the CD20-negative neoplastic cells (compare 5D to 5A). A higher magnification of the region circled in black in Figure 5D revealed strong consistent gB staining in essentially every blastoid tumor cell (Figure 5E and inset), similar to that seen in the lymph node tumor (Figure 7D). The A01112 colonic tumor was also tested with the EBNA-2 monoclonal antibody to determine whether there was a co-infection with LCV. As shown in Figure 5F, the A01112 tumor section showed discrete EBNA-2 staining in isolated cells scattered throughout the tumor (black circle). The staining appeared to be both cytoplasmic and nuclear (Figure 5G). Additional EBNA-2 staining was detected in the adjacent normal colonic mucosa (Figure 5F, red circle). This staining was very specific to the nuclei of distinct cells flanking the epithelial cells lining the mucosal crypts (Figure 5H and insert), possibly enterochromaffin-like cells. Figure 6G shows the nodular tumor in 99091 stained with the 1508 anti-gB antibody. Very strong gB staining was observed in CD20-negative/CD3-negative tumor (compare Figure 6A and 6G). Higher magnification of the region circled in black (Figure 6G) revealed strong cytoplasmic gB staining of the blastoid tumor cells (Figure 6I). In contrast, the small densely packed CD20-positive lymphocytes encircling the tumor did not stain for gB (Figure 6G (circled in red) and Figure 6H (higher magnification)). In all three CD3-negative/CD20-negative lymphomas, very high RV2 viral loads (median = 120 RV2 genomes/cell) correlated with expression of RV2 ORF59 and gB antigens indicating that the malignant lymphoma cells were infected with an RV2 rhadinovirus that was undergoing lytic replication. The low levels of LCV detected in these lymphoma samples (see Table 1) correlates with scattered EBNA-2 positive cells present in the adjacent normal tissue or infiltrating the tumor mass, as shown in Figures 5F–H. Interestingly, this level of LCV infection detected by immunohistochemistry in only a very limited number of cells in the A01112 lymphoma was below the level of detection of our sensitive LCV QPCR.

#### CD20-positive lymphomas

Previous studies have shown that a large percentage of SIV-associated B-cell lymphomas infected with LCV show a type III stage of latency characterized by expression of EBNA-2 or LMP-1 [27]–[29], [32]. Available antibody reagents to EBV LMP-1 do not appear to crossreact to LCV LMP-1 homologs [29], therefore, we examined the CD20-positive B-cell lymphomas that showed high LCV levels for expression of EBNA-2 using a monoclonal antibody to EBV EBNA-2 that crossreacts with the LCV EBNA-2 homologs. The A07762 lymphoma, which had nine LCV copies/cell, was negative for EBNA-2 staining in multiple tumor sections (data not shown). Similarly, A01110 lymphoma, which had 0.6 LCV copies/cell, was also negative for EBNA-2 staining (data not shown). However, the A08044 lymphoma with four LCV copies/cell stained strongly for EBNA-2 (Figure 4D), and this staining overlapped that of CD20 (compare Figure 4A and D). Higher magnification demonstrated strong EBNA-2 staining in the nuclei of essentially all of the tumor cells (Figure 4F). This staining was specific for the tumor cells, as the CD20-positive lymphocytes in the normal colonic lymphoid follicle did not stain for EBNA-2 (Figure 4D (circled in red) and 4E). We also examined the LCV-high CD20-positive lymphoma samples for expression of lytic antigens using a monoclonal antibody to the EBV viral capsid antigen (VCA). No staining was detected in any of the lymphomas, indicating a lack of LCV lytic replication (data not shown).

## Discussion

We analyzed a panel of macaque lymphomas collected at the WaNPRC over the past 30 years with the goal of elucidating the role of macaque gammaherpesviruses in simian AIDS-related lymphomagenesis. In previous published studies, macaque LCV homologs of EBV were detected in the vast majority of lymphomas by non-quantitative PCR techniques, with detection rates ranging from 75–100% [24], [25], [28], [31], [32], [40]. In our study, we used QPCR to quantitate viral loads in order to distinguish tumor-associated from incidental virus present in the lymphoma tissue section. Using a QPCR assay to detect macaque LCV homologs, our study showed that the macaque lymphomas segregated into three distinct groups. The first group (5/14 lymphomas) had high LCV loads with a median of four LCV genomes/cell (range 0.6–25). The second group (3/14 lymphomas) had low viral loads with a median of 0.02 LCV genomes/cell (range 0.005–0.06). The third group (6/14) lymphomas had no detectable LCV. Thus, less than half of the lymphomas in our study had a viral load consistent with widespread LCV infection of the tumor cells. A semi-quantitative study at the TNPRC in rhesus macaques found similar results, with high LCV viral loads detected in only 60% of SIV-associated lymphomas, and low or undetectable levels of LCV detected in the remainder [24].

To determine whether the lymphomas contained another macaque gammaherpesvirus, we used QPCR to detect and quantitate the levels of the macaque RV1 and RV2 Old World primate rhadinoviruses related to KSHV. First, we examined the lymphomas for the presence of RFHV, the macaque RV1 homolog of KSHV, since KSHV is associated with several B-cell disorders [5]. Using a highly sensitive QPCR assay, we have previously shown that RFHV is present at high viral loads (∼2 RFHV genomes per cell) in retroperitoneal fibromatosis, a KS-like tumor in macaques, paralleling the level of KSHV in KS tumors [37]. In the current study, however, we were unable to detect RFHV in any of the macaque lymphoma samples. Second, we examined the macaque lymphomas for the presence of a member of the more distantly-related RV2 rhadinovirus lineage. Although RRV, the RV2 lineage prototype in rhesus macaques, has been previously detected in macaques with SIV-associated hyperplasias and lymphomas, an etiological association was not supported or was unclear [23], [33], [34]. Our QPCR analysis detected RV2 rhadinoviruses, including RRV and homologs in pig-tailed and cynomolgus macaques (MneRV2 and MfaRV2, respectively), in twelve of the fourteen macaque lymphomas. As with LCV, the lymphomas segregated into three distinct groups. The first group (8/14 lymphomas) had high RV2 loads with a median of 70 RV2 genomes/cell (range 2–800). The second group (4/14) had low RV2 loads with a median of 0.004 RV2 genomes/cell (range 0.0007–0.04). The third group (2/14) had undetectable levels of RV2. Thus, more than half of the lymphomas in our study had levels of RV2 consistent with widespread infection within the lymphomas.

A comparison of the LCV and RV2 QPCR data showed that the lymphomas with high levels of LCV had low or undetectable levels of RV2 rhadinovirus and, conversely, those with high levels of RV2 had low or undetectable levels of LCV. These results suggest that LCV and RV2 have distinct, non-overlapping roles in the etiology of these lymphomas. Previous studies of macaque LCV in SIV-associated lymphomas have used either a probe to the latent EBV EBER RNA or an antibody to the EBV latency protein EBNA-2 to identify virus-infected cells. EBER RNA is a general marker of EBV latency, while EBNA-2 is a specific marker of the EBV latency program III [41]. In previous studies, LCV EBNA-2 expression was detected in approximately 60% of macaque lymphomas with variable numbers of positive tumor cells in each lymphoma. The frequency of LCV EBER RNA in these cases was generally higher [24]–[32], [40]. Of the five lymphomas in the LCV-High group of lymphomas in our study, only three (A07762, A08044 and A01110) had tissue blocks for immunohistochemical localization studies. Using a monoclonal antibody to the EBV EBNA-2 latency-associated protein, we observed strong staining in the A08044 lymphoma in the nuclei of the vast majority of the blastoid tumor cells. This is similar to previous results obtained for a subset of the SIV-associated lymphomas [27], [32], [42]. The other two LCV-High lymphomas were not reactive with the EBNA-2 antibody, as has been seen in a number of lymphomas in the studies above. Phenotype analysis revealed that two of the LCV-high lymphomas, A01110 and A08044, stained strongly for the B-cell marker CD20, confirming previous correlations between CD20 expression and EBV-associated lymphomas [27]. A08044 was also tested for expression of the B-cell antigen, BLA.36 and heterogenous staining was detected within the tumor sample (Table 1). The third lymphoma, A07762, within the LCV-high group showed heterogenous expression of CD20 within the tumors. An additional lymphoma from macaque 02206, which had an intermediate level of LCV (0.06 LCV genomes/cell), stained strongly for CD20. Like the LCV-high lymphomas, the 02206 lymphoma had a higher level of LCV than RV2 (∼10 fold), however, this lymphoma showed vast regions of necrosis that may have affected the viral load determination. The fifth LCV-high lymphoma, L758, obtained from the TNPRC, was not tested for CD20 reactivity, but had been positive for the B-cell antigen, BLA.36 in a previous study [33]. Thus, our study showed a strong correlation between high LCV viral loads and B-cell antigen expression in the SIV/SHIV-associated macaque lymphomas.

We detected high loads of RV2 rhadinovirus in all of the SRV-2-associated CD3-positive lymphomas and the SIV/SHIV-associated CD20-negative/CD3-negative lymphomas. This correlated with the strong expression of RV2 lytic antigens in the vast majority of the blastoid tumor cells, supporting a strong etiological association with these lymphomas. The median viral load for RV2 in the RV2-High group (∼70 RV2 genomes/cell) was nearly 20 fold higher than the viral load for LCV in the LCV-High group (∼4 LCV genomes/cell). Previous quantitative studies of the EBV load in human B-cell lymphomas detected viral loads of 1–15 EBV genomes/cell in EBER-positive latently-infected cells [11]. Consistent with this, we detected a viral load of ∼5 EBV genomes/cell in homogeneous cultures of latently-infected Raji cells *in vitro* (unpublished results) and four LCV genomes/cell in the LCV-high macaque lymphomas *in vivo*. Although we only detected the LCV latency marker, EBNA-2, in one of the three lymphomas tested, previous studies have shown a strong correlation between expression of latency markers, such as EBNA-2 and EBER LCV and the development of LCV-associated lymphomas [25]–[32], [40], [42]. We were unable to determine whether our macaque lymphomas expressed LMP-1, a major marker of EBV latency, due to the lack of reagents that cross react with the LCV homologs of the macaque species studied [29]. None of the lymphomas showed evidence of expression of the VCA lytic antigen. In contrast, the high RV2 rhadinovirus viral load (average = 130 RV2 genomes/cell), detected in the macaque lymphomas is similar to the viral load detected during lytic replication of RRV in cultured rhesus primary fetal fibroblasts (170 viral genomes/cell) [39]. Similar to the lytic replication of RRV detected in vitro in the previous study, the RV2-infected tumor cells in the lymphoma lesions in the present study expressed the ORF59 DNA polymerase processivity factor, an early marker of lytic replication, and the virion gB, a late marker of lytic replication. Thus, the high RV2 viral load in the RV2-High group of macaque lymphomas correlated with the RV2 lytic gene expression suggesting that the RV2 rhadinovirus was replicating in the tumor cells.

Extensive viral replication is not commonly found in tumors associated with an underlying viral infection [43]. The viral replicative stage is usually associated with cell lysis and death, as is evident in *in vitro* culture systems. Furthermore, virus replication is associated with the expression of the full panoply of viral proteins necessary for replication and virion assembly, all of which are targets of the immune system. Thus, susceptibility to cell death and immune control would seem to be counterproductive for a viable tumor virus. In previously characterized tumor viruses, such as KSHV or EBV, virus latency is a common feature of the virus-infected tumor cell, as the virus is able to minimize its perturbation of cellular homeostasis and evade the immune response by limiting the expression of viral proteins [43]. In our study, large areas of necrosis were detected in many of the macaque lymphomas however, there was little evidence of lysis of individual RV2-infected tumor cells expressing lytic antigens in the lymphoma lesions. The large size of the tumors detected in these macaques and their associated pathology indicate that the RV2 gene expression and the levels of lytic replication detected in our study were insufficient to limit the spread of these tumor cells. Interestingly, while the majority of KS tumor cells are latently infected with KSHV, a small proportion of cells show markers of lytic replication. Since most of the KSHV proteins implicated in tumorigenesis, including vGPCR, vIL-6, and vBCL-2 are lytic cycle genes not expressed during latency, it is believed that lytic cycle gene expression is critical for KS pathogenesis [44]. Further study is ongoing into the nature of the replicative infection present in the macaque tumors.

While the high levels of both LCV and RV2 detected in the lymphomas in our study are compatible with a widespread infection of the tumor cells in the respective lymphomas, the low levels of each virus detected in other lymphomas suggests the presence of incidental virus or virus-infected normal cells in the tumor lesion. In support of this conjecture, we detected occasional EBNA-2-positive cells in the tumor mass and in adjacent non-tumor epithelial cells in the RV2-High/LCV-Low lymphomas. Thus, the low levels of LCV in these lymphomas were not tumor-associated but, instead, were incidental in the tumor lesion. Similarly, RV2 antigen expression was occasionally detected in normal lymphoid follicles and in non-tumor epithelial cells adjacent to the tumor mass in LCV-High/RV2-Low tumors. This indicated that the low RV2 load in these samples was not tumor-associated, but instead was also due to incidental virus in the tumor section. Nearly all of the macaque lymphomas analyzed contained detectable levels of both LCV and RV2. However, quantitation studies demonstrated that only one of these viruses in each lymphoma was present at a level that was compatible with an etiologic or causative role. These results indicate that care must be taken when making an assertion of virus-association based on simple PCR or limited immuohistochemistry analysis.

It has previously been reported that RRV, the rhesus macaque RV2 rhadinovirus, was associated with non-Hodgkin's lymphoma in one of two macaques experimentally infected with RRV and one of fifteen macaques co-infected with RRV and SIV [34]. In this study, RRV DNA was detected in tissue samples by PCR but no quantitation was reported. The RRV genome was detected in CD20-positive B-cells in one SIV-associated, LCV-negative lymphoma, and in CD20-negative/IgM+ B-cells in an SIV-negative lymphoma using *in situ* hybridization. RNA transcripts for vIL6, ORF71, ORF72 and ORF73 were detected in the CD20-positive lymphoma. All four of these RRV genes are expressed during RRV replication [45], [46], in contrast to KSHV where the gene homologs are expressed during latent infection. These results suggest that the rhesus lymphomas in this study, like the lymphomas in our study, are infected with an RV2 rhadinovirus that is in a replicative stage.

Five of the macaque lymphomas typed as CD3+ T-cell lymphomas. All five were from pig-tailed macaques and were associated with an SRV-2 infection that had been endemic in the WaNPRC macaque colony prior to 1990. Interestingly, the SRV-2 epidemic was also associated with a high frequency of retroperitoneal fibromatosis, a KS-like tumor. While previous studies revealed high levels of RFHV in the SRV-2 associated retroperitoneal fibromatosis lesions [37], [47], we were unable to detect RFHV in any of the SRV-2 associated T-cell lymphomas. Instead, high viral loads of RV2 rhadinoviruses were detected in these lymphomas. This was coupled with the strong expression of the ORF 59 DNA polymerase processivity factor in the nucleus and the virion glycoprotein gB in the cytoplasm of the lymphoma cells, indicating that the neoplastic CD3-positive T-cells were infected with RV2 and were replicating the virus. Herpesvirus saimiri, a rhadinovirus of New World monkeys related to KSHV, causes acute T-cell lymphoma in non-native monkey species [48]. Although there have been anecdotal reports of KSHV-associated T-cell lymphomas [6], or KSHV-associated lymphomas mimicking T-cell lymphomas [49], our study is the first to report a T-cell tumor syndrome associated with a rhadinovirus infection in its natural host. Current studies are delineating the differentiation stage of these cells.

Our data support an etiological role for both macaque lymphocryptovirus and RV2-lineage rhadinoviruses in simian AIDS-associated lymphomas in multiple macaque species. We found a strong association between macaque LCV and B-cell lymphomas expressing CD20. We also found a strong association between macaque RV2 rhadinoviruses and CD20-negative/CD3-negative and CD20-negative/CD3-positive lymphomas. Our results indicate that the two different macaque gammaherpesviruses related to EBV and KSHV infect and have pathogenic roles in different lymphocyte lineages and differentiation stages. Furthermore, whereas macaque LCV, like the human gammaherpesviruses EBV and KSHV, are latent in the proliferating tumor cells, the RV2 rhadinoviruses express both early and late stage replication markers and show high viral loads consistent with ongoing virus replication. This suggests that these gammaherpesvirus lineages have profoundly different interactions with their target host cells and with the host immune system.

Our data are consistent with roles for both lymphocryptoviruses and RV2 rhadinoviruses in the etiology of simian AIDS-associated lymphomas. Interestingly, studies have shown that less than half of AIDS-related B-cell lymphomas in HIV-infected individuals are associated with high levels of EBV [11]. Our data suggests that the remainder of these lymphomas could be associated with an undiscovered human homolog of the macaque RV2 rhadinoviruses. We are currently developing reagents and assays to detect this putative human rhadinovirus.

## Materials and Methods

### Clinical evaluation and tissue sampling from breeding colony and experimental animals

Archived tissue samples were obtained from both genders of different species of macaques diagnosed with malignant lymphoma at the WaNPRC over the past thirty years. All animals were euthanized for clinical reasons and complete necropsies were performed. Diagnosis of lymphoma was based on gross and histopathologic examination. Tissues collected at necropsy were fixed in 10% neutral buffered formalin and embedded in paraffin. Additional samples were obtained from a cynomolgus macaque from C. Emerson, Lovelace Respiratory Research Institute (Albuquerque, NM). This animal was diagnosed with lymphoma during quarantine. In addition, DNA samples from a previously characterized lymphoma from an SIV-infected rhesus macaque [33] was obtained from L. Levy, Tulane National Primate Research Center, (Covington, LA).

### DNA extraction

Formalin-fixed paraffin-embedded tissue was treated with xylene to remove paraffin, followed by extensive ethanol washes. DNA was extracted from embedded and frozen tissues using standard proteinase K-phenol/chloroform extractions and concentrated by ethanol precipitation.

### Real-time QPCR

Viral loads were determined using real-time QPCR with TaqMan primers and probes. The RV1 and RV2 rhadinovirus assays have been described previously [36], [37]. The LCV assay amplifies a 188-bp amplicon from the DNA polymerase genes of EBV, MneLCV, MfaLCV, and MmuLCV using consensus primers “LCVa” (forward primer 5′-GCCACCACATGCCCTT-3′) and “LCVb” (reverse primer 5′- CCTAAGACCTAATAAAGGCC-3′) with a TaqMan probe “LCV-FAM” 5′-(6-FAM)- CAAGGAGTACCTGCGTCTCATTC -(BHQ-1)-3′. Viral copy number per cell was determined using a QPCR assay targeting oncostatin M (OSM), a single copy cellular gene [36]. The copy number for each assay was calculated from the cycle threshold (C_t_) using the Bio-Rad software. The viral load was determined as a cellular genome copy equivalent by using the formula:
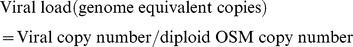
Samples were assayed in duplicate and the means were determined. Standard deviations were calculated using the sum of the errors of the viral and OSM copy number determinations.

### Antibodies

Various polyclonal and monoclonal antibodies were developed in-house or purchased. 1508 – Pan anti-RV1/RV2 glycoprotein B rabbit polyclonal antiserum: The 1508 antiserum was produced by immunization of a rabbit with a peptide N-TVFLQPVEGLTDNIQRYFSQ-C that is present in the glycoprotein B of the human (KSHV) and macaque RV1 and RV2 rhadinoviruses (unpublished results). 425 – anti-RV2 ORF59 rabbit polyclonal antiserum: The 425 antiserum reacts with macaque RV2 ORF59 homologs but not with macaque RV1 or LCV homologs of ORF59 [39]. Rabbit-anti human-CD3 and mouse anti-human CD20 were purchased from DAKO (Carpinteria, CA). The A27–42 mouse IgG3 antibody to the B lymphocyte antigen 36 (BLA.36) was obtained from BioGenex (Fremont, CA) and was shown to crossreact with the macaque homolog of the B-cell antigen. Additional BLA.36 immunolabeling was performed on the lymphoma from macaque L758 in a previous study [33]. Biotinylated goat anti-rabbit-Ig and horse anti-mouse-IgG were purchased from Vector Laboratories (Burlingame, CA). The mouse monoclonal PE2 antibody to EBV EBNA-2 was purchased from Abcam (San Francisco, CA) The mouse monoclonal OT15e antibody to EBV BFRF3, the p18 small viral capsid antigen (VCA) [50] was obtained from J. Middeldorp. The goat anti-human IgM labeled with HRP was purchased from Southern Biotech (Birmingham, Al).

### Immunohistochemistry

Archival tissue samples were deparaffinized, subjected to antigen retrieval by heating in appropriate buffer solutions, and incubated with primary antibody. Bound antibody was visualized with biotinylated secondary antibody and preformed avidin-biotin horseradish peroxidase complexes with the chromagen diamino-benzidine or 3-amino-9-ethyl-carbazole [47]. Sections were counterstained with hematoxylin, examined on a Nikon Eclipse E600 microscope, photographed using a Nikon Coolpix 5300 camera or imaged using an Olympus NanoZoomer Digital Pathology Microscope, and prepared for publication using Adobe Photoshop (CS).

### Accession numbers/ID for genes and proteins

*Lymphocryptovirus* genus: Human herpesvirus 4/Epstein-Barr virus (HHV4/EBV; YP_401712) and macaque lymphocryptovirus of *M. mulatta* (MmuLCV; AAK95475), *M. nemestrina* (MneLCV; unpublished data) and *M. fascicularis* (MfaLCV; AF534221); *Rhadinovirus* genus, RV1 lineage of Old World primate rhadinoviruses: human herpesvirus 8/Kaposi's sarcoma-associated herpesvirus (HHV8/KSHV; AAC57086) and macaque retroperitoneal fibromatosis herpesviruses of *M. mulatta* (RFHVMm; AAC57976), *M. nemestrina* (RFHVMn; AAF81662), and *M. fascicularis* (RFHVMf; AAN35122); and the RV2 lineage of Old World primate rhadinoviruses: macaque RV2 rhadinoviruses of *M. mulatta* (RRV; NP_570750), and *M. fascicularis* (MfaRV2; ABU52895).

## Supporting Information

Dataset S1 **Detailed pathology reports for the macaque lymphoma cases.**
(DOC)
