# Supplementary material for: Macaque Homologs of EBV and KSHV Show Uniquely Different Associations with Simian AIDS-related Lymphomas
Source: PLoS Pathog. 2012 Oct 4;8(10):e1002962. doi: 10.1371/journal.ppat.1002962 (PMC3464224; doi:10.1371/journal.ppat.1002962)
Supplement: Dataset S1 — Detailed pathology reports for the macaque lymphoma cases. (DOC) [file ppat.1002962.s001.doc]

**Lymphoma Cases**

**CD3-positive**

**T76088:** *M. nemestrina*, female, 6.8 years old. SRV2-positive WaNPRC breeding colony animal diagnosed with wasting syndrome, generalized lymphadenopathy and suspect lymphosarcoma. Gross necropsy revealed acute severe multifocal malignant lymphoma in lymph nodes and multiple visceral organs and tissues, including spleen, liver, kidney and lung. Histologically the neoplastic cells are large, with abundant cytoplasm and a large nucleus with vesiculated chromatin and a prominent nucleolus. The lymphomas were classified as lymphoblastic and were phenotypically CD3 positive.

**T76321:** *M. nemestrina*, male, 5.8 years old. SRV2- and SIV-positive WaNPRC breeding colony animal diagnosed with inflammation of the lung and lymph node hypertrophy. Gross necropsy revealed acute severe multifocal malignant lymphoma in multiple visceral organs, including liver, spleen, kidney, pancreas, lymph nodes, lung, adrenal gland, urinary bladder, salivary gland, and bone marrow. The lymphomas were classified as lymphoblastic and were phenotypically CD3 positive.

**T80120:** *M. nemestrina*, female, 2.5 years old. Suspect SRV2-infected WaNPRC breeding colony animal diagnosed with inflammation of the lung. Gross necropsy revealed acute severe multifocal malignant lymphoma in multiple visceral organs, including thymus, spleen, bone marrow, lymph nodes, kidney, liver, and adrenal gland,. Histologically the thymus-associated neoplasms consisted of sheets of lymphoblastic cells and smaller non-cleaved, intermediately differentiated lymphocytes. Neoplastic lymphocytes infiltrated the interstitium of the kidneys, sinuses of the liver, the adrenal cortices as well as the bone marrow. The lymphomas were classified as lymphoblastic and were phenotypically CD3 positive

**T81497:** *M. nemestrina*, male, 10.9 years old – SRV2-positive WaNPRC breeding colony animal diagnosed with intestinal inflammation and wasting syndrome. Gross necropsy revealed acute severe multifocal malignant lymphoma in liver, kidney, stomach, colon, lymph nodes, pancreas, spleen, bone marrow and salivary gland. Histologically the tumor nodules were composed of large blastoid cells demonstrating marked anisokarrhyosis and anisocytosis. They had oval to angular nuclei with a large central, magenta nucleolus, dense, dispersed chromatin and abundant cytoplasm. Amongst the blastoid cells were scattered bi- and multi-nucleated cells and bizarre, Reed-Sternberg-like cells. The lymphoma was classified as anaplastic large cell lymphoma with Reed-Sternberg-like cells, and the neoplastic lymphoid cells, including the RS-like cells were phenotypically CD3 positive.

**T81228:** *M. nemestrina*, male, 9.7 years old. SRV2-positive WaNPRC breeding colony animal diagnosed with wasting syndrome and severe oral necrosis. Gross necropsy revealed acute severe multifocal malignant lymphoma in multiple visceral organs, including spleen, lymph nodes, liver, and bone marrow. Histologically the neoplasms presented as sheets and clusters of large, pleomorphic cells with round, oval or irregularly shaped nuclei. Scattered throughout the neoplastic cells are large bi- and multi-nucleated cells, resembling Reed-Sternberg cells. The lymphomas classified as anaplastic large cell lymphoma with Reed-Sternberg cells and were phenotypically CD3 positive.

**CD3-negative/CD20-positive**

**02206:** *M. nemestrina*. Female, 14.7 years old, SHIV-positive. At necropsy, the abdominal cavity contained 25-30 ml of cloudy, serosanguinous ascites, and the omentum was adherent to various organs in multiple foci. Located in and along the greater curvature of the stomach was a very firm, elongated (approximately 5 x 6 x 8 cm) mass extending from the stomach wall and involving the pancreas, which was not discernable. The gastric mucosa was necrotic in a ~ 5 cm in diameter area involving the cardia and fundus, in direct extension of the neoplasm. The stomach content was dark-brown and contained coagulated blood. On cut surface the neoplasm appeared as consisting of reddish marbled lobules or nodules separated by septae of dense fibrous tissue, and multifocally there were foci of necrosis with caseation. The stomach was partly adherent to the liver, which was mildly enlarge, yellowish marbled and very friable with slightly rounded edges. The liver bulged slightly through the diaphragm along the esophagus (herniation). The tracheobronchial and mediastinal lymph nodes were completely effaced by large conglomerates of neoplasms with a hemorrhagic appearance, and multiple areas of necrosis on cut surfaces. Histologically, the masses were composed of dense sheets of small to medium-sized, rarely large, lymphoblastoid cells supported by a delicate vasculo-reticular stroma. The lymphoid cells had round to ovoid nuclei with dispersed to finely granular chromatin and a single magenta, acentrically located nucleolus. The cytoplasm was scant to moderately abundant, eosinophilic and with distinct cell borders. Mitoses were frequent (> 6/ hpf) and sporadically bizarre. Multifocally there were variably sized areas of necrosis, sporadically accompanied by hemorrhage. Some areas of the masses in the lymph nodes contain large number of macrophages with large phagosomes, giving the field a “starry sky” appearance. In the gastric mucosa, pancreas and liver the neoplastic cells appear to spread by invasion, and there is extensive tissue necrosis and effacement of normal histological architecture of the tissues. The neoplasms were diagnosed as centroblastic lymphoma based on cell morphology.

**L758:** *M. mulatta*. Male, 2.5 years old. SIV-positive. SAIDS-associated immunoblastic lymphoma described by {Ruff, 2003 #1618}.

**95017:** *M. fascicularis*. Male, 4.5 years old, SRV2-negative, SIV-positive. No available gross or histopathological descriptions.

**A01110:** *M. mulatta*. Male, 8.7 years old. SRV2-negative, SIV-positive, Terminated due to a severe intractable gastro-enteritis and other SAIDS-defining symptoms. At necropsy the animal was found to have a severe colitis, severe proliferative enteropathy and hydropericardium. Several small nodular proliferations were noted in the gastric (fundic) mucosa or submucosa, but the epithelium appeared intact. Histologically these nodules were composed of neoplastic lymphocytes with an appearance as described for A01112,

**A08044**: *M. fascicularis*. Female, 7 years old, SRV2-negative, infected with RT-SHIVtc. The animal developed chronic thrombocytopenia, and anemia, weight loss, and anorexia. A large abdominal mass was identified that was adhered tightly to the cranial pole of the right kidney, the pancreas, the fundic and pyloric stomach, duodenum and proximal jejunum. Sections of the abdominal mass revealed sheets, nests and cords of moderate to usually large-lymphoblastic lymphocytes with mostly cleaved nuclei, moderate to high mitotic index with moderate numbers of irregular mitotic figures, and there was scant to occasionally abundant/robust bands of fibrous stroma. The neoplasm appeared to originate from the duodenal lamina propria where there was effacement thereof and protrusion and multifocal ulceration of distended mucosa. From here the neoplasm exhibited transmural infiltration and effacement with extension into mesentery with incorporation/obliteration of some mesenteric nodes, and there were areas of moderate to extensive infiltration of pancreatic ducts and glands with adjacent pancreas exhibiting atrophy, degeneration and interstitial fibrosis, the right adrenal gland was completely enveloped by the neoplasm with slight infiltration through the capsule, and throughout the neoplasm there was mild to moderate, granulomatous inflammation. The cranial pole of the right kidney also had a large region of infiltration by the neoplasm with regional effacement of parenchyma, with displacement and compression of adjacent parenchyma, and connective tissue of the pelvic region had moderate numbers of neoplastic cells. The left ventricular freewall and the interventricular septum also had metastatic foci of neoplastic cells that are moderate to large with myocellular displacement/effacement and some areas of myocellular degeneration.

**A07762:** *M. fascicularis*, Female 2.4 years old, SRV2-negative, STLV-1 negative. The animal showed no signs of disease other than bilateral enlargement of the axillary lymph nodes and a unilaterally enlarged inguinal lymph node. The lymph node enlargement throughout the body was remarkable at necropsy.

**CD3-negative/CD20-negative**

**93034:** *M. fascicularis*. Female, 11 years old. SRV2-negative, experimentally infected with SIV/PBJ14 uncloned stock infected was euthanatized due to clinical deterioration and fulfillment of SAIDS-criteria. At necropsy multiple (>15) subcutaneous firm, tan-white neoplasms were found. Additional neoplasms were found in the myocardium, the cervical and uterine wall, both ovaries, pancreas, liver, omentum and retroperitoneal interstitae, thymus and many lymph nodes. Histologically, the neoplasms were composed of infiltratively expanding sheets of heterogenous, lymphoblastoid cells, 15-30 μm in diameter, with an often very large, mostly round nucleus with finely dispersed or vesiculated chromatin, a large central or acentric magenta nucleolus, surrounded by moderate to abundant eosinophilic cytoplasm and distinct cell borders. In all affected tissues this was accompanied by degenerative changes in normal tissue constituents, including the myocytes of the myocardium, uterus and cervix, follicles in the ovaries, acinar cells in the pancreas and hepatocytes and bile ducts in the liver. In most of the neoplasms there was widespread hemorrhage and necrosis, both of the neoplastic cells and of the affected tissue proper. The histomorphological appearance of the neoplastic lymphocytes is compatible with follicular large cell lymphoma.

**99091:** *M. fascicularis,* Male 9 years old. SRV2-negative. A well-demarcated nodular growth was present in the cortico-medullary zone of the left kidney and bilaterally the normal adrenal structure was partially effaced by similar well-demarcated tan-white neoplasms. Histologically these neoplasms were composed of sheets of lymphoblasts with moderate to scant cytoplasm, round nuclei with granular chromatin and mostly one central, magenta nucleolus. The mitotic frequency varied from 1 to 3 per high-power filed (40X objective). Similar neoplastic lymphocyte infiltrates were found in the cauda equine, the myocardium, and the right kidney. Other findings in this animal included a severe meningo-encephalomyelitis, polyneuritis, interstitial nephritis, and fungal eosophagitis. The lymphomas were classified as lymphoblastic.

**A01112:** *M. mulatta*, male, 5.5 years old. A WaNPRC animal experimentally infected with SIVsmE66 who spontaneously died after showing anorexia and lethargy. At necropsy the inguinal and axillary lymph nodes were enlarged, and associated with the right axillary lymph nodes was a large, well-circumscribed, non-infiltrative and firm neoplasm with several prominent blood vessels running deep into tissue. A similar growth (2x2 cm in diameter) was present on in the abdomen partly infiltrating the muscle-layer at or near the navel. Multiple additional tumors were present: on the capsule of the left kidney, in the proximal mesentery, in and on the intercostal muscles, in the diaphragm, and on the pericardium; all of a similar appearance: firm, well-circumscribed, whitish on cut surface with variable necrosis and hemorrhage. The ileocecal orifice was swollen, ulcerated with a small blood-clot lightly adherent to the edge. Located in the cardia and fundic region of the stomach were multiple firm, approximately 1 cm in diameter, round nodules with an indented center surrounded by a zone of hemorrhages and hyperemia. These neoplasms were associated with an area of approximate 3-4 cm in diameter where the mucosa was diffusely eroded and ulcerate, bordered by thickened, hemorrhagic edges. A small, 5-8 mm in diameter, firm, nodular growth was present on the posterior serosal surface of the bladder. A further 5 nodules were present on the mucosal surface. Light microscopic examination showed that with minor variations dependent on the tissue localization all neoplasms were well-demarcated, partly encapsulated masses composed of sheets of large (10-15μm) pleomorphic and anisocytotic lymphoid cells with distinct cell-borders, moderate to abundant dense eosinophilic cytoplasm, large round to angular nuclei (anisokarrhyosis) with vesiculated or finely dispersed chromatin and 1-3 large magenta nucleoli either centrally located or adjacent to the nuclear membrane. The neoplastic cells were supported by a fine fibrovascular stroma with no or only rare large vessels. Variable, but mostly large proportions of the neoplasms were necrotic (intravital change), and throughout the viable areas were many singly scattered cells with karrhyorectic and karrhyolytic nuclei. Mitoses were frequent (3-4/hpf) and often bizarre. This presentation is compatible with large cell or immunoblastic lymphoma.
